# Effects of Cystamine on antioxidant activities and regulatory T cells in lupus-prone mice

**DOI:** 10.1111/jcmm.12107

**Published:** 2013-08-02

**Authors:** Tsai-Ching Hsu, Chun-Ching Chiu, Yi-Wen Wang, Bor-Show Tzang

**Affiliations:** a Institute of Microbiology and Immunology, Chung Shan Medical UniversityTaichung, Taiwan; b Clinical Laboratory, Chung Shan Medical University HospitalTaichung, Taiwan; c Graduate Institute of Basic Medical Science, China Medical UniversityTaichung, Taiwan; d Department of Healthcare Administration, Asia UniversityTaichung, Taiwan; e Department of Neurology and Department of Medical Intensive Care Unit, Chunghua Christian HospitalChunghua, Taiwan; f Institute of Biochemistry and Biotechnology, Chung Shan Medical UniversityTaichung, Taiwan; g Department of Biochemistry, School of Medicine, Chung Shan Medical UniversityTaichung, Taiwan

**Keywords:** Systemic lupus erythematosus (SLE), Cystamine, regulatory T cells, antioxidant

## Abstract

Attenuated antioxidant activities, irregular cytokines expressions and reduced regulatory T cells, are strongly associated with the pathogenesis of systemic lupus erythematosus (SLE). Despite the well-established beneficial effects of cystamine on lupus-prone mice, the extent to which cystamine contributes to antioxidant activity and the reduction of regulatory T cells has seldom been investigated. Therefore, this study elucidates how cystamine affects anti-oxidant activities in NZB/W F1 mice by performing assays of Glutathione (GSH), 1,1-diphenyl-2- picryl-hydrazyl (DPPH) and malondialdehyde thiobarbituric acid (MDA). In addition, investigations of the effects of cystamine on CD4^+^/CD25^+^ regulatory T cells and interleukin-6 (IL6)/STAT-3 signalling were performed with flow cytometry and immunoblots. Experimental results reveal more significantly reduced MDA and increased GSH and DPPH in NZB/W F1 mice receiving cystamine than in those mice receiving PBS. Meanwhile, CD4^+^/CD25^+^ regulatory T cells more significantly increase in NZB/W F1 mice receiving cystamine than in those mice receiving PBS, accompanied by significantly reduced IL-6/phosphorylated STAT-3 expression. The above findings suggest the beneficial effects of cystamine in terms of increasing antioxidant activities and CD4^+^/CD25^+^ regulatory T cells in lupus-prone mice by suppressing IL-6/STAT3 signalling.

## Introduction

Systemic lupus erythematosus (SLE) is an autoimmune disease with an unknown aetiology [1]. According to numerous studies, excessive formation of reactive oxygen species (ROS) has a higher risk of inflicting damage on lipids, proteins and DNA [2, 3]. Such formation is also linked to various autoimmune diseases, including SLE. Further evidence suggests that elevated oxidative stress and free radical–associated reactions play important roles in SLE progression and pathogenesis [4–6].

According to a previous study, lipid peroxidation level significantly increased in SLE patients, whereas anti-oxidant enzyme activities like those of super oxide dismutase (SOD), catalase (CAT), glutathione peroxidase (GPx) and anti-oxidant molecule glutathione (GSH) more significantly reduced in SLE patients than in the control cases [7–9]. In addition, oxidative modification in proteins has been shown to elicit antibodies in SLE, including oxidatively modified DNA (8-oxodeoxyguanine) and low-density lipoproteins (LDL) [7, 10]. Actually, the autoantibodies in the sera of SLE patients exhibited a significant enhanced reactivity against catalase and SOD-modified proteins [11]. Meanwhile, a recent study has associated dysregulation of T cells in SLE with elevated oxidative stress. Evidence further suggests that lipid peroxidation-derived aldehydes contribute to trichloroethene-mediated autoimmunity and irregular Th1 cells activation in SLE [12]. Another study noted a lower frequency of regular T cells in New Zealand Black/White (NZB/W) F1 lupus-prone mice than in non-autoimmune strains [13]. Similar results were also observed in SLE patients with active disease [14]. These findings strongly suggest a connection between oxidative stress and the generation of autoimmunity in SLE.

Cystamine can interfere with transglutaminase 2 (TG2) activity by forming mixed disulphide [15, 16]. As is well known, cystamine is highly promising for use as an anti-oxidant [17] and anti-apoptotic compound [18, 19], as demonstrated by its widespread use in neuron protection [20, 21]. Our recent studies have demonstrated the beneficial effects of cystamine on SLE by reducing the expression of inflammatory related proteins and serum titre of anticardiolipin (aCL) autoantibodies in NZB/W F1 mice as well as alleviating the abnormalities in various organs such as the brain, heart and liver. Conversely, Balb/C mice receiving PBS did not significantly differ from those receiving cystamine [22–27]. Owing to the relatively little knowledge of how cystamine affects anti-oxidant activity and regulatory T cell distribution in SLE, this study investigates how cystamine contributes to antioxidant activity and CD4^+^/CD25^+^ regulatory T cells in NZB/W F1 mice.

## Materials and methods

### Mice and cystamine treatment

Female NZB/W F1 mice, a well-described lupus-prone mice strain [28], were purchased from animal centre, National Taiwan University, Taiwan and housed under supervision of the Institutional Animal Care and Use Committee at Chung Shan Medical University. Treatment of cystamine is described elsewhere [22]. Mice at age of 16-week were divided into two groups (10 mice/group) and given PBS or cystamine treatment for experiments. For cystamine (Sigma-Aldrich, Saint Louis, Mo, USA) treatment, NZB/W F1 mice were injected intraperitoneally (i.p.) with 100 μl of 10 mM phosphate buffer saline (PBS) or 100 μl of 10 mM cystamine daily for 28 days. Heart blood sample was collected and serum was stored at −80°C until use. The organs were harvested into appropriate storage buffers before further analysis.

### Measurement of glutathione levels

Determination of glutathione levels is described elsewhere [29]. Measurements of concentrations of reduced glutathione GSH were performed with GSH assay kit (Chemicon Inc., Temecula, CA, USA) according to the manufacturer's protocol. In brief, the measurement at 380/461 nm of 90 μl of protein extracts and 10 μl of prepared monochlorobimane solution incubated for 2 hrs at 25°C away from light and fluorescence was performed with a 96-well fluorometric plate reader.

### DPPH assay

The radical scavenging activity was determined *via* a 1,1-diphenyl-2-picryl-hydrazyl (DPPH) scavenging activity as described elsewhere [30]. Briefly, a solution of 180 μl of 0.1 mM DPPH solution in ethanol was gently mixed with 20 μl sample in ethanol. The value of DPPH absorption was measured at 517 nm by a 96-well fluorometric plate reader. 1,1-diphenyl-2- picryl-hydrazyl radical scavenging activity was expressed as% inhibition compared to the blank (ethanol).

### Thiobarbituric acid reductase assay

Lipid peroxidation is measured *via* thiobarbituric acid reductase (TBAR) assay as described elsewhere [31]. Briefly, to make a 10% homogenate, a volume of 200 mg serum or tissue was added to 2 ml of 1.15% KCl and mixing was performed with a homogenizer (Knotes Glass, Vineland, NJ, USA). A quantity of 3 ml of 1% phosphoric acid and 1 ml of 0.6% TBA solution were added to 0.5 ml of 10% tissue homogenate. The mixture was left in a boiling water bath for 45 min. After cooling, 4 ml of n-butanol was added and mixed vigorously. The butanol phase was separated by centrifugation at 500 × *g*, and absorbance was measured spectrophotometrically at 535 nm. The difference was used as the TBAR value, and the results were calculated as nanomoles per gram of tissue.

### Flow cytometry analysis of T cell populations

Mice were killed as described above and the spleens were harvested and placed in cold RPMI-1640 medium supplemented with 10% foetal bovine serum (Invitrogen, Carlsbad, CA, USA), 10 mg/ml gentamycin, 2 mM L-glutamine, and 0.1 mM 2-mercaptoethanol. Red blood cells were lysed in red blood cell lysing buffer (Biolegend, San Diego, CA, USA) on ice according to manufacturer's instruction. Splenocytes were stained with optimal concentrations of fluorochrome conjugated mAbs (10^6^ cells in 200 μl of phosphate-buffered saline, 1% bovine serum albumin and 0.1% sodium azide) and fixed with 1% paraformaldehyde. Monoclonal antibodies against CD3 with FITC conjugated, CD4 with FITC or PE conjugated, CD8 with FITC conjugated and CD25 with PE conjugated were used for analysis of T cell markers (BD Pharmingen, Franklin Lakes, NJ, USA). For CD4^+^/CD25 regulatory T cells purification, a CD4^+^CD25^+^ Regulatory T Cell Isolation Kit was used according to the manufacturer's instructions (Miltenyi Biotec, Bergisch Gladbach, Germany). Samples were analysed on a FACSCalibur instrument (Becton Dickinson, Mountain View, CA, USA) and stored at −80°C for further immunoblotting assay.

### Expression of Foxp3 mRNA

Extraction of total cellular mRNA from 1 × 10^6^ isolated cells was performed with Dynabeads® mRNA DIRECT™ (Invitrogen) and reverse transcription was performed with SuperScript TM II reverse transcriptase (Invitrogen). Quantification of gene expression by real-time PCR was performed with Assays-on-Demand TM gene expression products [forkhead box P3 (Foxp3) and hypoxanthine phosphoribosyltransferase (HPRT)] and the ABI/PRISM 7700 sequence detection system (Applied Biosystems Inc., Foster City, CA, USA). Three independent experiments were performed. The relative expression of Foxp3 was determined by dividing Foxp3 gene value by the HPRT value.

### Enzyme-linked Immunoabsorbent Assay

Detection of Anti–dsDNA antibodies and Anti-nuclear antibodies (ANA) levels in serum was performed with a RayBio mouse Anti–dsDNA ELISA kit (INOVA Diagnostic Inc., San Diego, CA, USA) and a BioAssay™ mouse ANA ELISA Kit (US Biological Inc., Salem, MA, USA) according to manufacturer's instructions. No significant variation in Anti–dsDNA antibodies and ANA levels was detected (Fig. S1) as described in our previous study [22].

### Haematoxylin—eosin staining

The kidney samples of animals were excised and soaked in formalin and covered with wax. Slides were prepared by deparaffinization and dehydration. They were passed through a series of graded alcohols (100%, 95% and 75%), for 15 min. each. The slides were then dyed with haematoxylin. After gently rinsing with water, each slide was then soaked with 85% alcohol, 100% alcohol I and II for 15 min. each. At the end, they were soaked with Xylene I and Xylene II. Photomicrography was performed with Zeiss Axiophot microscopes and photomicrographs were obtained.

### GBM thickness and Immunoglobin deposition

Kidney tissue sections were prepared as described above. For measurement of glomerular basement membrane (GBM) thickness, three tissue blocks of kidney cortex from each mouse were analysed. The full thickness of the GBM was measured on a strictly perpendicular section on at least 10 sites of six capillary loops, giving a total of 60 measurements per mouse. For immunoglobin deposit assay, tissue sections were incubated with peroxidase-conjugated rabbit antimouse immunoglobins (anti-total Igs, DAKO Corp., Carpinteria, CA, USA). The peroxidase activity was visualized with diaminobenzidine. Determination of the positive signal areas of 10 areas, with each containing one glomerulus, was performed with Leica Qwin Standard V2.6 (Leica Microsystems, Welzlar, Germany). Three tissue sections of kidney cortex from each mouse were analysed for measurements of GBM thickness and immunoglobin deposition.

### Immunoblotting

Sodium dodecyl sulphate-polyacrylamide gel electrophoresis (SDS-PAGE), was performed with 12.5% acrylamide gel, as previously described [32]. The protein samples were homogenized sufficiently with B25 High-shear Dispersing Emulsifiers Homogenizing machine (BRT CO, Shanghai, China) and centrifuged at 13,400 × *g* in 4°C for 30 min. Supernatants were isolated and denatured for 5 min. in boiling water with sample buffer (0.0625 M Tris–HCl buffer, pH6.8, containing 2.3% SDS, 5% 2-mercaptoethanol and 10% glycerol). Samples applied to the gel were run of 100–150 V for 90 min. and electrophoretically transferred to nitrocellulose membrane (Amersham Biosciences, Piscataway, NJ, USA). The membrane was then soaked in PBS with 5% non-fat dry milk for 30 min. at room temperature to saturate irrelevant protein binding sites. Antibodies against IL-6, STAT-3, phosphorylated STAT-3, PI3K, phosphorylated-PI3K and β-actin (Upstates, Charlottesville, VA, USA; Chemicon Int.) were diluted in PBS with 2.5% BSA and incubated for 90 min. with gentle agitation at room temperature. The membranes were washed twice with PBS–Tween for 60 min. and secondary antibody conjugated with horseradish peroxidase (HRP) was added. Pierce's Supersignal West Dura HRP Detection Kit (Pierce Biotechnology Inc., Rockford, IL, USA) was used to detect antigen–antibody complexes. The blots were scanned and quantified by densitometry (Appraise, Beckman-Coulter, Brea, CA, USA).

### Statistical analysis

All the statistical analyses were performed with SPSS 10.0 software (SPSS Inc, Chicago, IL, USA). Three independent experiments were repeated. Statistical analyses were performed with the Student's *t*-test and one-way anova. *P* < 0.05 was considered statistically significant.

## Results

### Cystamine increases the expression of anti-oxidant enzymes in NZB/W F1 mice

**Fig. 1:**
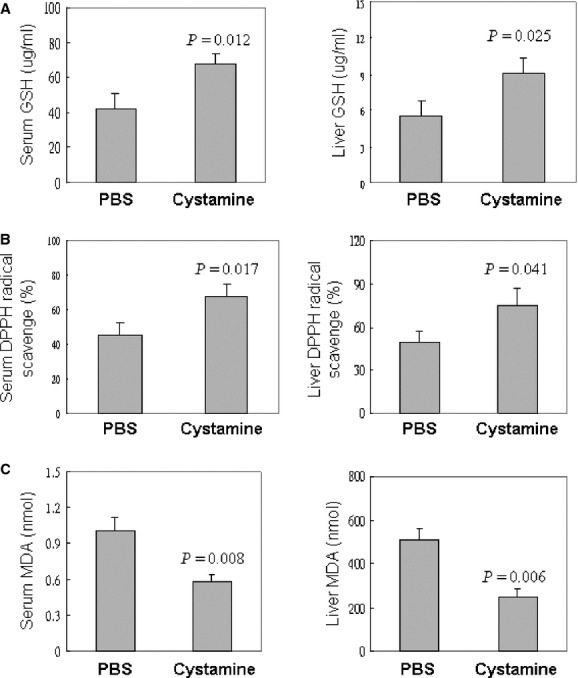
Effects of cystamine on (**A**) GSH, (**B**) DPPH and (**C**) MDA levels in serum and liver of NZB/W F1 mice. Data are means ± SD of 10 animals in each group. A *P* < 0.05 was considered to be statistically significant. PBS: phosphate buffer saline; GSH: Glutathione; DPPH: 1,1-diphenyl-2-picryl-hydrazyl; MDA: malondialdehyde thiobarbituric acid.

1,1-diphenyl-2- picryl-hydrazyl (a free radical scavenger) and MDA (an abundant aldehyde reacting with lysine residues by forming Schiff bases) [33] have been associated with the oxidative stress and pathogenesis in SLE [34]. This study examined how cystamine affects anti-oxidant activities in SLE by detecting the levels of GSH, DPPH and MDA in both serum and livers from NZB/W F1 mice. According to our results, GSH and DPPH levels in both serum and liver samples from NZB/W F1 mice receiving cystamine more significantly increased than in those mice receiving PBS (Fig. 1A and B). Conversely, levels of serum and liver MDA more significantly reduced in NZB/W F1 mice receiving cystamine than in those mice receiving PBS (Fig. 1C).

### Effects of cystamine on kidney architecture changes and immunoglobin deposition in NZB/W F1 mice

**Fig. 2:**
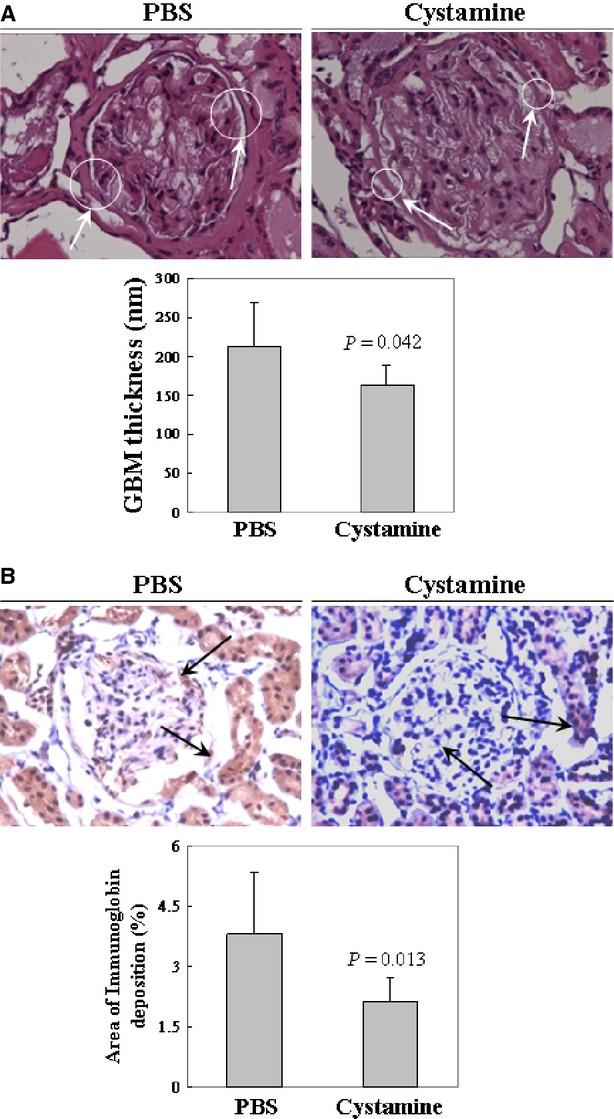
Kidney histopathological changes in NZB/W F1 mice. Histopathological analysis of kidney tissue sections with haematoxylin and eosin staining from NZB/W F1 mice. The images of kidney architecture were magnified by 400 times and (**A**) the glomerular basement membrane (GBM) thickening was indicated by arrows. Lower panel indicated the measurement of full GBM thickness. (**B**) The Immunoglobin deposition is indicated by arrows and the positive signal area is shown in the lower panel. A *P* < 0.05 was considered to be statistically significant. PBS: phosphate buffer saline; GBM: glomerular basement membrane.

This study also examined how cystamine affects kidney architectures in NZB/W F1 mice by performing histopathological analysis on kidney tissue stained with haematoxylin and eosin. Notably, GBM thickening and immunoglobin deposition more significantly reduced in Malpighian corpuscle of kidney from NZB/W F1 mice receiving cystamine than in those mice receiving PBS (Fig. 2).

### Cystamine increases the expression of CD4^+^/CD25^+^ regulatory T cell in NZB/W F1 mice

**Fig. 3:**
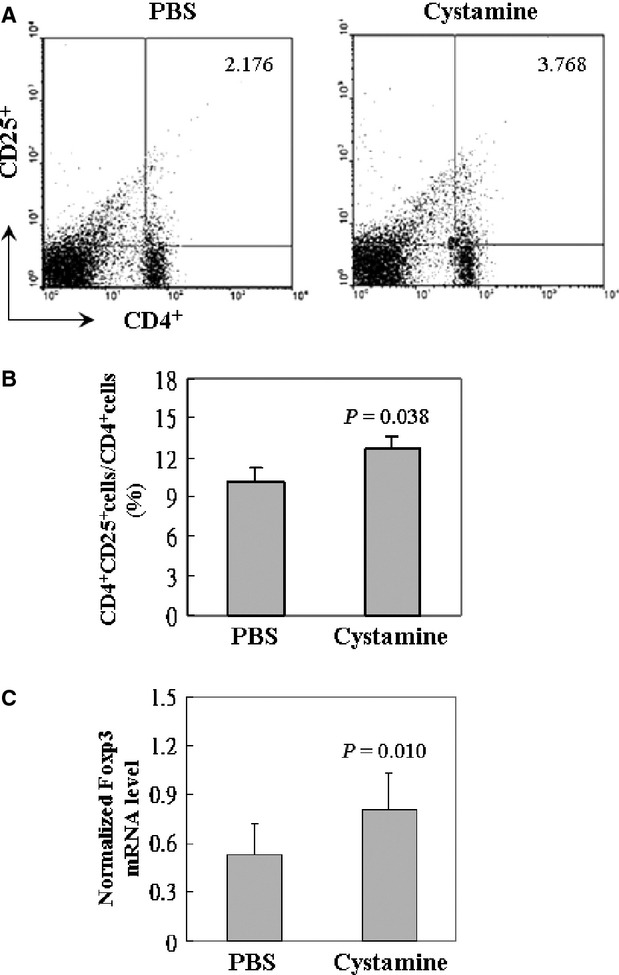
The relative percentage of splenic CD4^+^CD25^+^ regulatory T cells in NZB/W F1 mice. The CD3^+^ T cells were gated and the percentage of CD25^+^ cells in CD4^+^ T cells was shown. Bars are means ± SD of 10 animals in each group. A *P* < 0.05 was considered to be statistically significant. PBS: phosphate buffer saline.

Moreover, this study examined how cystamine affects regulatory T cells by determining the percentage of CD4^+^CD25^+^ T cells in the CD4^+^ T cells from NZB/W F1 mice treated with PBS or cystamine. Figure 3A shows a representative finding of flow cytometry indicating the percentage of CD4^+^CD25^+^ T cells in whole spleen cells. In addition, the mean percentages of CD4^+^CD25^+^ T cells from NZB/W F1 mice receiving PBS and cystamine were 2.176% and 3.768% respectively. The relative percentages of splenic CD4^+^CD25^+^ T cell in NZB/W F1 mice receiving PBS or cystamine were determined (Fig. 3B). The percentage of CD4^+^CD25^+^ regulatory T cells in splenic CD4^+^ T cells of NZB/W F1 mice receiving cystamine was significantly higher than in those mice receiving PBS; in addition, the mean percentages were 10.79 ± 0.63% and 12.386 ± 0.88% respectively. Meanwhile, a significantly increased mRNA level of Foxp3 was detected in CD4^+^CD25^+^ T cell in NZB/W F1 mice receiving cystamine than in those mice receiving PBS (Fig. 3C).

### Cystamine attenuates the IL-6 expression and STAT-3/PI3K signalling

**Fig. 4:**
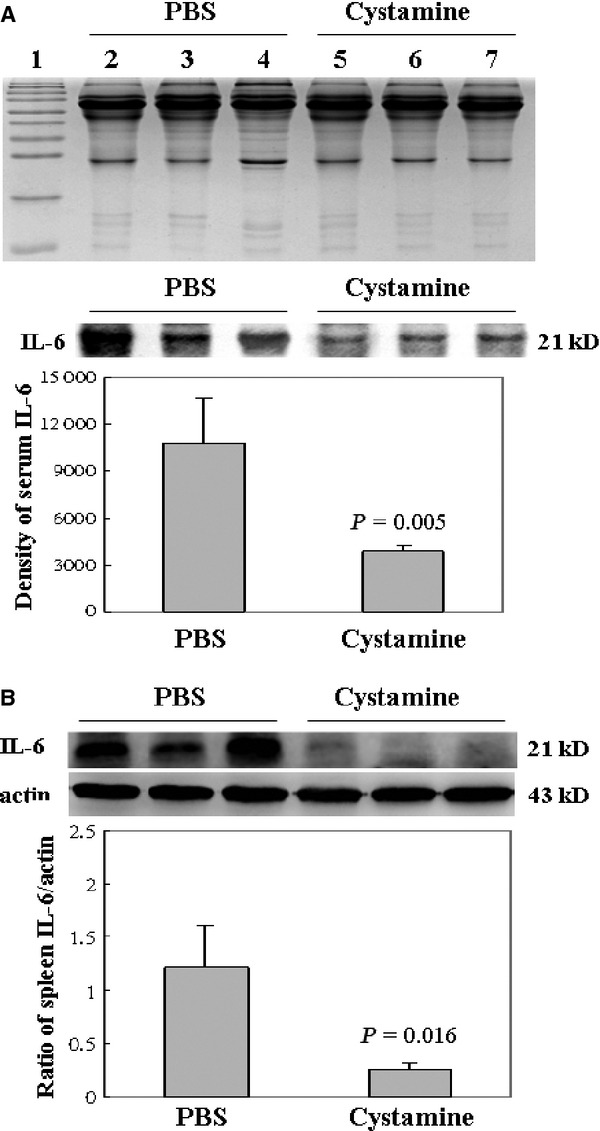
Effects of cystamine on IL-6 expression in (**A**) serum and (**B**) CD4^+^CD25^+^ regulatory T cells from NZB/W F1 mice. The upper panel of A is the loading control of serum sample. Similar results were obtained in three independent experiments. A *P* < 0.05 was considered to be statistically significant. PBS: phosphate buffer saline; IL-6: interleukin-6.

**Fig. 5:**
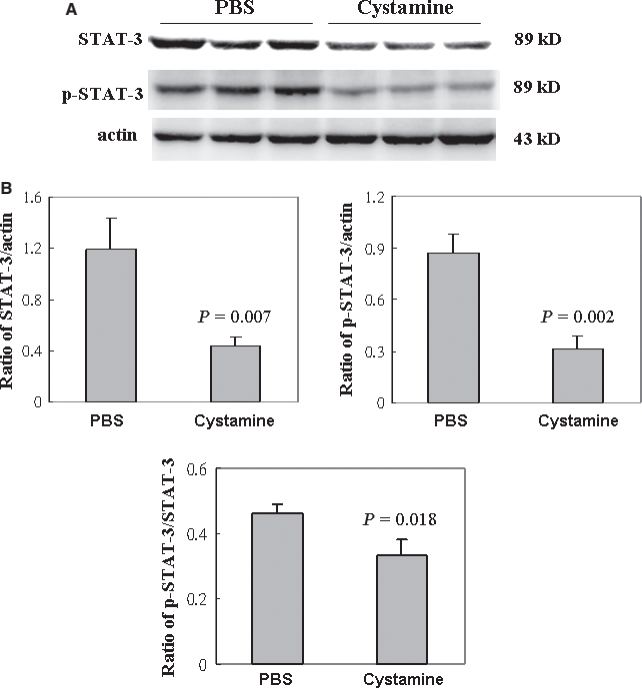
Effects of cystamine on STAT-3 protein expression in CD4^+^CD25^+^ regulatory T cells from NZB/W F1 mice. Expression of STAT-3 and phosphorylated STAT-3 proteins were detected by immunoblottings. Similar results were obtained in three independent experiments. A *P* < 0.05 was considered to be statistically significant. PBS: phosphate buffer saline; STAT-3: Signal Transducer and Activator of Transcription 3.

**Fig. 6:**
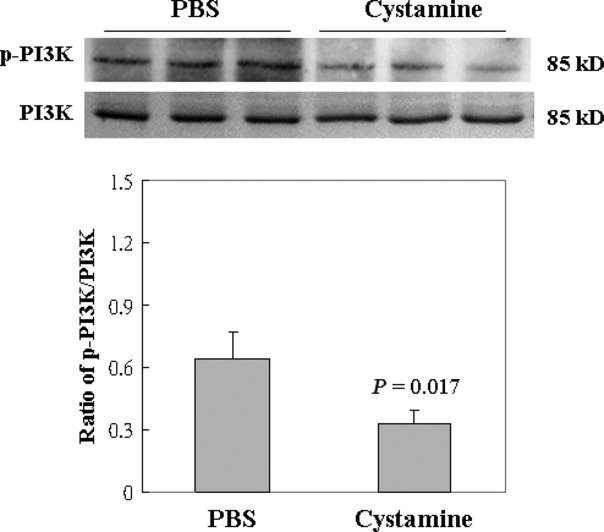
Effects of cystamine on PI3K protein expression in CD4^+^CD25^+^ regulatory T cells from NZB/W F1 mice. Expression of PI3K and phosphorylated PI3K (p-PI3K) proteins were detected by immunoblottings. Similar results were obtained in three independent experiments. A *P* < 0.05 was considered to be statistically significant. PBS: phosphate buffer saline; PI3K: Phosphatidylinositide 3-kinases.

Elevated IL-6 levels in both murine models and SLE patients were detected and have been associated with the decline of anti-oxidant activities and regulatory T cells. This study examined how cystamine affects IL-6 expression by performing immunoblots. Experimental results indicated that IL-6 level was more significantly reduced in the serum of NZB/W F1 mice receiving cystamine than in those mice receiving PBS (Fig. 4A). Meanwhile, the presence of IL-6 was more significantly decreased in CD4^+^CD25^+^ regulatory T cells from NZB/W F1 mice receiving cystamine than in those mice receiving PBS (Fig. 4B). Exactly how cystamine influences IL-6 signalling in SLE was more closely examined by studying the presence of STAT-3 and its phosphorylated form. According to those results, the ratio of phosphorylated STAT-3 (p-STAT-3)/STAT-3 was more significantly reduced in CD4^+^CD25^+^ regulatory T cells from NZB/W F1 mice receiving cystamine than in those mice receiving PBS (Fig. 5). Moreover, the phosphorylation of PI3K, a downstream molecule of STAT-3, was also examined. Experimental results indicated that the ratio of p-PI3K/PI3K was more significantly decreased in CD4^+^CD25^+^ regulatory T cells from NZB/W F1 mice receiving cystamine than in those mice receiving PBS (Fig. 6).

## Discussion

Significantly reduced activities of anti-oxidant enzymes [9] and decline of regulatory T cells have been identified in SLE patients and associated with the generation of autoantigens [8, 10] and autoantibodies [35]. Despite the well-established beneficial effects of cystamine on SLE, exactly how cystamine contributes to antioxidant activity and reduced regulatory T cells remains unclear. This study has demonstrated significantly increased antioxidant activities, including elevated GSH and DPPH levels and reduced MDA in NZB/W F1 mice receiving cystamine. Meanwhile, CD4^+^/CD25^+^ regulatory T cells significantly increased in NZB/W F1 mice receiving cystamine by reducing IL-6/STAT-3 signalling.

Accumulated oxidative stress implies that oxidation process could form adducts with proteins and significantly enhance their immunogenicity [8]. Indeed, a higher systemic lupus erythematosus disease activity index (SLEDAI) score is positively associated with the elevated oxidative stress in patients with SLE [6]. A related study has demonstrated that various oxidative proteins, including oxidized low-density lipoproteins (LDL) and 8-oxoeoxyguanine, cause premature atherosclerosis in SLE patients [8, 36]. Immunization with 4-hydroxy-2-nonenal modified 60 kD Ro autoantigen induces an accelerated epitope spreading in an animal model of SLE [10]. In addition, low intracellular GSH pools are implicated in defective T cell function in SLE [8]. As various antioxidant activities are markedly reduced in SLE [7–9], enhancement of antioxidants is viable for preventing or ameliorating oxidative damage in SLE. In this study, GSH and DPPH levels in NZB/W F1 mice receiving cystamine more significantly increased than in those mice receiving PBS. In addition, MDA level was more significantly reduced in NZB/W F1 mice receiving cystamine than in those mice receiving PBS. The above findings demonstrate the feasibility of increasing antioxidant activities in NZB/W F1 mice by administering cystamine.

As a multifunctional cytokine, interleukin-6 (IL-6) plays important roles in the pathogenesis of SLE. In this study, elevated level of IL-6 was detected in both human and animal models with SLE [37–40], as well as it contributed to the suppression of regulatory T cells [41]. Obviously, a lower number and functions of T-regulatory cells were identified in lupus-prone animal models and in SLE patients [13, 14, 42]. However, the blockade of interleukin-6 by anti-IL-6 monoclonal antibodies treatment could improve the dysfunction of T cells in SLE [43]. In addition, a recent study further demonstrated that IL-6-mediated loss of Treg suppression requires phosphorylation of Stat3 [41]. These studies demonstrated that IL-6/STAT-3 signalling significantly contributes to the loss of functional suppression of regulatory T cells [41]. Notably, according to our results, the CD4/CD25 positive regulatory T cell more significantly increased in the spleens of NZB/W F1 mice receiving cystamine than in those mice from the control group. Meanwhile, IL-6/STAT-3 signalling more significantly reduced in the spleens of NZB/W F1 mice receiving cystamine than in those mice from the control group. The above findings demonstrate that cystamine up-regulates the population of CD4/CD25 positive regulatory T cells by suppressing IL-6/STAT-3 signalling pathway.

## Conclusions

Cystamine, an inhibitor of TG2 [15, 16], has beneficial effects on various diseases such as neurodegeneration and SLE [20]. Our recent study has demonstrated how cystamine alleviates the inflammation and apoptosis in brain, liver and heart of NZB/W F1 mice [22–27]. Importantly, this study demonstrates the beneficial effects of cystamine on inducing antioxidant activities and CD4^+^/CD25^+^ regulatory T cells in lupus-prone mice by decreasing IL-6/STAT3 signalling. Our results further demonstrate that cystamine is highly promising for its use in treating SLE.

## Supporting information

**Figure S1** Effects of cystamine on serum level of (A) Anti–dsDNA antibodies and (B) Anti-nuclear antibodies in NZB/W F1 mice. Similar results were obtained in three independent experiments. A *P* < 0.05 was considered to be statistically significant. PBS: phosphate buffer saline; dsDNA: double strand DNA; ANA: antinuclear antigen.
