# Corrigendum

**DOI:** 10.1111/jcmm.12258

**Published:** 2014-02-27

In [1], The third and fourth affiliations of this paper (^c^Graduate Institute of Basic Medical Science, China Medical University, Taichung, Taiwan, and ^d^Department of Healthcare Administration, Asia University, Taichung, Taiwan) should be deleted and the affiliations of the first author (Tsai-Ching Hsu) should be corrected as follows:

Tsai-Ching Hsu:

^a^Institute of Microbiology and Immunology, Chung Shan Medical University, Taichung, Taiwan

^b^Clinical Laboratory, Chung Shan Medical University Hospital, Taichung, Taiwan

The authors wish to apologize for any misunderstanding or inconvenience caused.
