# Supplementary figures and images for: Effects of Cystamine on antioxidant activities and regulatory T cells in lupus-prone mice
Source: J Cell Mol Med. 2013 Aug 2;17(10):1308–15. doi: 10.1111/jcmm.12107 (PMC4159022; doi:10.1111/jcmm.12107)

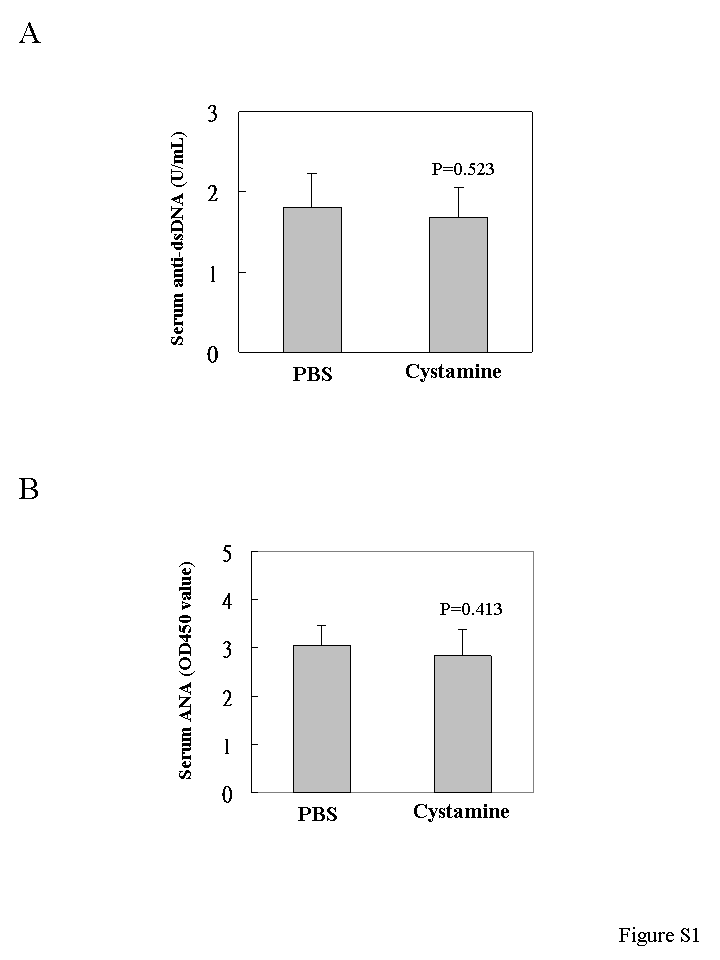

Supplement: Supplementary file 1 [file jcmm0017-1308-SD1.tif]
